# Association between exposure to traumatic events and anxiety disorders in a post-conflict setting: a cross-sectional community study in South Sudan

**DOI:** 10.1186/1471-244X-14-6

**Published:** 2014-01-10

**Authors:** Touraj Ayazi, Lars Lien, Arne Eide, Leslie Swartz, Edvard Hauff

**Affiliations:** 1 Institute of Clinical Medicine, Faculty of Medicine, University of Oslo, PO Box 1171, Blindern, 0318 Oslo, Norway; 2 National Center for Dual Diagnosis, Innlandet Hospital Trust, Furnesvegen 26, 2380 Brumunddal, Norway; 3 SINTEF Technology and Society, PO Box 124, Blindern, 0314 Oslo, Norway; 4 Alan J. Flisher Centre for Public Mental Health, Department of Psychology, Stellenbosch University, Private Bag X1, 7602 Matieland, South Africa; 5 Division of Mental Health and Addiction, Department of Research and Development, Oslo University Hospital, Ulleval, Kirkeveien 166, Building 20, 0407 Oslo, Norway; 6 Faculty of Public Health, Hedmark University College, PO Box 400, 2418 Elverum, Norway

**Keywords:** PTSD, Anxiety disorders, Trauma exposure, Post-conflict, South Sudan

## Abstract

**Background:**

The negative effect of exposure to traumatic events on mental health is well known. Most studies of the effects of trauma on mental health in war-affected populations have focused on post-traumatic stress disorder (PTSD) and depression. Although some studies confirm the existence of anxiety symptoms in war-affected populations, the extent to which exposure to traumatic events is independently associated with anxiety diagnoses (other than PTSD) has received less attention. The study aimed to determine whether having an anxiety diagnosis, other than PTSD, was associated with experiencing traumatic events in a post-conflict setting, across genders and after controlling for demographic and socio-economic variables.

**Methods:**

In this cross-sectional community study (n = 1200), we applied the Harvard Trauma Questionnaire (HTQ) to investigate the extent of trauma exposure and PTSD. The Mini-International Neuropsychiatric Interview (MINI) was used to investigate the prevalence of anxiety disorders: generalized anxiety disorder (GAD), panic disorder (PD), social phobia, obsessive-compulsive disorder (OCD), and agoraphobia. Multinomial logistic regression analyses were conducted to examine the association between these disorders, previous trauma exposure, and socio-economic factors.

**Results:**

The participants were 56.4% male and 43.6% female. The age ranged between 18 and 73 years old (Mean 34.63, SD = 12.03). The estimated rates of GAD-only and PD-only (without comorbidity with PTSD) were 5.5% and 3.1%, respectively. Exposure to traumatic events and socio-economic disadvantage were significantly associated with having one or more anxiety diagnoses. After controlling for age, sex, rural/urban setting, and socio-economic disadvantage, exposure to trauma was independently associated with anxiety diagnosis. There were gender differences in the pattern of risk factors for having PTSD, GAD or PD.

**Conclusion:**

In individuals with a history of war-related trauma exposure, attention should be given to symptoms of GAD and PD, in addition to PTSD symptoms.

## Background

A large body of research documents the association between exposure to armed conflict-related traumatic events and negative mental health outcomes such as post-traumatic stress disorder (PTSD), depression, and anxiety [1-4]. Much of this research has focused on PTSD and depression [5-7], with anxiety disorders studied to a lesser extent. The current study aimed to examine the association between exposure to traumatic events and anxiety disorders in a post-conflict setting.

A limited number of studies, mostly conducted in high-income countries, have shown associations between trauma exposure and various anxiety disorders. Ghafoori et al. [8] reported an association between September 11 traumatic events and generalized anxiety disorder (GAD) symptoms among adult patients seeking primary care treatment in the USA. Elevated rates of GAD have also been associated with exposure to traumatic events among natural disaster-affected populations [9-12] and motor-vehicle accident survivors [13]. Several studies of victims of terrorist attacks [14] and victims of natural disasters [11] have documented the association between panic disorder (PD) and exposure to traumatic events. Sexual and physical abuse are traumatic events that have also been considered a risk factor for panic disorder [15] in a patient population of Cambodian refugees [16].

A few studies have investigated the experience of anxiety among conflict-affected populations; these have identified exposure to traumatic events as a risk factor for a higher degree of anxiety symptoms [17,18]. However, most of these studies have focused on anxiety *symptoms* and their association with history of traumatic exposure, rather than examining anxiety *diagnoses* as the outcome. One exception is De Jong et al.’s study [6] where mental health consequences of war in several countries were investigated. The results showed elevated levels of anxiety disorders, PTSD, and depression. Anxiety disorder was however defined as a category of disorders encompassing panic disorder, agoraphobia, and social and specific phobia. In another study of war-affected communities in five Balkan countries, higher rates of anxiety disorders were associated with more potentially traumatic experiences during and after the war [19]. Similarly to the latter study, ‘anxiety disorder’ was considered to be any combination of anxiety disorders (PD, agoraphobia, social phobia, obsessive-compulsive disorder, PTSD, or GAD).

There is increasing interest in understanding the mental health consequences of traumatic events beyond PTSD [20-23]. Despite the relevance of this issue to research and interventions in post-conflict settings [24-26], there has been little research on this topic.

Being female has been identified in several studies as a risk factor for developing PTSD, depression, and anxiety symptoms in conflict-affected settings [27-30]. Most of the available knowledge about sex differences in the outcome of exposure to trauma comes from PTSD studies. Several epidemiological studies from post-conflict settings report a higher prevalence of PTSD among women than men [30,31]. However, the gender differences in PTSD may be attributed to differences in the types of trauma experienced rather than to gender itself. Women are likely to experience particular types of traumatic events: a review study suggested that women are more likely to experience sexual assault than men [32]. In the study by de Jong et al., of four post-conflict countries, the rates of PTSD among men and women differed across countries [4]. Women had more PTSD symptoms than men in both Cambodia and Algeria, similar rates to men in Ethiopia, and fewer PTSD symptoms than male counterparts in Gaza. There is little knowledge about sex differences in particular anxiety disorders and their association with traumatic events in post-conflict settings.

The current study draws on data from a community study of the mental health of the population of South Sudan, which is one of the most economically disadvantaged countries in the world and has extremely scant health facilities [33]. In addition to economic hardship, the country has experienced more than 20 years of armed conflict. The signing of the Comprehensive Peace Agreement in 2005 ended extensive war-related violence and large-scale forced displacement and resulted in the creation of the new state of South Sudan in 2011. Despite this positive pattern of change, the growing influx of returnees to South Sudan has placed an extraordinary strain on already scant services and resources. The few studies conducted among the South Sudanese population show high levels of trauma exposure and psychological distress [34], including a PTSD rate of 37.6% [35].

The current study aimed to:

• estimate the prevalence rates of PTSD, and other anxiety disorders (GAD, PD, social phobia, OCD, and agoraphobia), and to estimate the prevalence rate of either GAD or PD without the presence of PTSD in male and female participants;

• examine the association between exposure to traumatic events and the above-mentioned anxiety disorders for male and female participants; and

• examine the levels of psychological distress associated with various anxiety disorders for male and female participants.

## Methods

A cross-sectional community survey was conducted in the Greater Bahr el Ghazal region of South Sudan in 2010. The Greater Bahr el Ghazal region consists of four states: Northern Bahr el Ghazal, Western Bahr el Ghazal, Lakes, and Warrap. It borders the Central African Republic to the west and Sudan to the north and has an estimated population of three million. A major part of the area is covered by swamps and ironstone plateaus. The region is populated by different ethnic groups, the main one being Dinka; other ethnic groups are Blanda, Jur/Lou, Nuer, Bari, and Zande [36]. The population in the region is predominantly rural with some variance across the four states; 92% of the population in Northern Bahr el Ghazal is classified as rural, compared with 57% in Western Bahr el Ghazal. Besides the official language of English, and Arabic, which is widely spoken in the region, Dinka, Blanda, Jur/Lou, Nuer, Bari, and Zande are the spoken indigenous languages [36,37].

The sample was drawn from the general population of the four states in the Greater Bahr el Ghazal region. A multistage random cluster sampling method was used. Nine randomly selected administrative units (‘Boma’) constituted the survey clusters, with a corresponding running cumulative population size for each Boma. The population data were based on the 2008 Sudan census [37]. These data were considered the most accurate population data available. In the next stage, the “spin-the-pen” method from the WHO Expanded Programme on Immunization [38] was used for household selection: the approximate geographic centre of the area was identified and one household along an imaginary line connecting the centre to the periphery was selected at random. Subsequent households were then selected by visiting every third-closest household. Within each selected household, individuals who were 18 years or older and gave informed consent to take part in the study were assigned a number. A card was drawn at random from a deck of cards with corresponding numbers, and the household member with that number was then interviewed. Individuals who were not able to or declined to give informed consent, or were visibly intoxicated were excluded from the study.

The interviewers were health personnel (n = 11, five women and six men) from the region who were familiar with the cultural traditions and fluent in the relevant local languages. They participated in two rounds of training workshops (nine days) prior to the data collection, during which they were trained in using the survey instruments, and the cultural acceptability of the interview protocol was discussed. The research instruments were available in both English and Arabic, but the main language used was Arabic, which is widely used in the area. In addition, the key terms of the questionnaire were discussed and translated into the indigenous languages of the area to ensure that the interviewers could easily explain all the items to the participants. Each household was approached by both a male and a female interviewer to ensure the interviewer’s gender would match that of the participant. If any psychopathology that required urgent treatment was identified amongst the participants, the interviewer referred the person to an associated health provider. A total of 1,236 households were contacted, from which 1,200 participants were recruited. The response rate was 95%.

Ethical clearance was obtained from the Research Department in the Ministry of Health of the Government of South Sudan and the Norwegian Regional Committee for Medical Research.

### Instruments

A questionnaire addressing socio-demographic factors, including sex, age, marital status, level of education, employment situation, monthly household income, and rural/urban setting, was administered to all participants. The questionnaire also included the Harvard Trauma Questionnaire (HTQ), which is a widely used instrument for assessing history of exposure to traumatic events and PTSD symptom criteria. The HTQ has been adapted for and used in various cultures and languages [39]. The Arabic version of the HTQ was employed in this study, after minor adaptations for the specific traumatic events in the South Sudan setting. The Arabic version of HTQ includes 40 questions on exposure to traumatic events with “Yes” or “No” answer choices. Participants were asked to confirm or disconfirm having been exposed to each of these 40 traumatic events: a) during the civil war (from 1983 to 2005) and b) after the Peace Agreement (after 2005). This gave us the opportunity to assess both recent and older traumatic experiences, which may also differ in character.

The HTQ also contains 40 questions aimed at identifying PTSD “caseness”. Participants were asked to report whether they had experienced the symptoms in question within the previous two weeks using a four-point scale: “0 = not at all”, “1 = a little”, “2 = quite a bit”, and “4 = extremely”. These first 16 questions in HTQ were derived from the (DSM-IV) criteria for PTSD [40]. To calculate scores on HTQ, we applied the DSM-IV algorithm which is developed by Mollica et al. [41] to identify caseness of PTSD. Positive response (more than or equal to 3) on the first 16 symptom questions [41] was calculated as follows:

– at least 1 of the 4 re-experiencing symptoms

– at least 3 of the 7 avoidance and numbing symptoms, and

– at least 2 of the 5 arousal symptoms.

The Mini-International Neuropsychiatric Interview (MINI) [42] (Arabic version) was used to detect anxiety disorders. More specifically, current GAD, PD, Obsessive-Compulsive Disorder (OCD), social phobia, and agoraphobia were assessed. The MINI is a structured diagnostic psychiatric interview instrument and has been translated and used in many languages, and applied in various cultures and settings [43].

The General Health Questionnaire (GHQ-28) was applied to measure the level of psychological distress. The GHQ-28 is a screening instrument widely used to detect psychological distress in community settings and non-psychiatric clinical settings [44]. It has been used in various populations and cultural settings [45], including Sudan [46]. Each item has a four-point severity scale (‘not at all’, ‘no more than usual’, ‘rather more than usual’ and ‘much more than usual’) with corresponding value of 0-1-2 or 3. For each participant, a total GHQ-28 score is calculated by adding the scores of each individual item. A higher total score on the GHQ-28 indicates more severe psychological distress (score range = 0–84) [47].)

Internal reliability was evaluated using Cronbach’s alpha. It was estimated to be 0.94 for GHQ-28 (psychological distress). Internal reliability for the HTQ-symptoms was estimated to be 0.75 (during the war) and 0.85 (after the Peace Agreement), which were above the commonly accepted level of 0.70 [48].

### Data analyses

Data analyses were conducted using SPSS (PASW) 20.0. Descriptive statistics were used to describe the characteristics of the sample and the extent of the four diagnostic categories: GAD-only, PD-only, PTSD-only, and “any anxiety disorder”. GAD-only, PD-only, PTSD-only were defined as presence of GAD, PD or PTSD, respectively, and absence of comorbidity with each other. For instance, a participant in GAD-only category had a GAD diagnoses alone without any comorbidity with PTSD or PD. The variable “any anxiety disorder” consisted of at least one of the following diagnoses (current): GAD, PD, OCD, social phobia, or agoraphobia, with no comorbidity with PTSD. Missing data were excluded from the analysis. For any given variable, the maximum amount of missing data was less than 5%.

Separate multivariate logistic regression analyses were applied to examine the association between the independent variables (socio-demographic variables and traumatic event exposure) and each of the dependent variables (having GAD-only, PD-only, PTSD-only, or “any anxiety disorder”). These analyses were conducted separately for men and women.

The traumatic event exposure variable consisted of both traumatic events during the war and after the Peace Agreement. The socio-demographic factors included; age, sex, marital status, rural/urban setting, income regularity, employment status, education level, household income, and being a returnee. We combined five conditions (having no regular income, low monthly income, unemployment, being widowed/separated/living in a polygamous marriage, and having no formal education) to create a variable of ‘socio-economic disadvantages’ in order to investigate the association between such disadvantages and anxiety disorders. This variable had three levels: “severely disadvantaged” (meeting four or five of the five conditions), “moderate disadvantaged” (meeting two or three of the conditions), and “mildly/not disadvantaged” (meeting one or none of the conditions).

For each separate hierarchic logistic regression analysis, the independent variables of age, rural/urban setting, and socio-economic disadvantage were entered into the analysis at the first step. At the second step, exposure to traumatic event during the war and after the Peace Agreement was entered. The severity of psychological distress, measured by GHQ-28 score, was compared among five different diagnostic categories: GAD-only, PD-only, and PTSD-only, “any anxiety disorder”, and those with “no anxiety disorder”. One-way ANOVA and post-hoc multiple comparisons, Tukey’s test was applied.

## Results

Table 1 shows the prevalence rates and socio-demographic characteristics of the participants across various anxiety disorders. The rates of anxiety disorders (without considering comorbidity) were as follows: GAD 15.8% (n = 189), PD 13.2% (n = 158), agoraphobia 17.8% (n = 212), social phobia 15.3% (n = 183), and OCD 12.7% (n = 151).

**Table 1:** Socio-demographic characteristics across different anxiety diagnoses

	**Total n (%)**	**GAD-only n (%)**	**PD-only n (%)**	**PTSD-only n (%)**	**Any anxiety disorder n (%)**
	1200 (100)	85 (7.1)	55 (4.6)	301 (25.5)	307 (26.0)
Sex					
Male	660 (56.4)	46 (7.0)	31 (4.7)	142 (21.9)	167 (25.7)
Female	510 (43.6)	36 (7.1)	19 (3.8)	154 (30.7)*	123 (24.4)
Urban/rural setting					
Urban	934 (77.8)	53 (12.1)	47 (5.1)	251 (27.3)	217 (23.6)
Rural	299 (22.2)	32 (5.7)*	8 (3.1)	50 (19.2)*	90 (34.4)*
Age (years)					
18–25	308 (26.0)	17 (5.6)	16 (5.2)	70 (23.2)	83 (27.3)
26–35	391 (33.1)	26 (6.6)	19 (4.9)	100 (26.2)	97 (25.4)
36–50	395 (33.4)	35 (8.9)	19 (4.9)	100 (25.4)	107 (27.2)
> 50	89 (7.5)	6 (6.7)	1 (1.1)	23 (26.1)	17 (19.3)
Socio-economic position					
Mildly disadvantaged	353 (37.2)	11 (3.1)	15 (4.3)	81 (23.7)	72 (21.0)
Moderately disadvantaged	503 (52.9)	49 (9.8)	24 (4.8)	122 (24.4)	145 (29.1)
Severely disadvantaged	94 (9.9)	6 (6.4)	2 (2.1)	45 (48.4.4)*	21 (22.6)*
		**Mean (95% CI)**			
Traumatic events during the war**	15.06 (14.50–15.56)	13.63 (11.73–15.52)	16.45 (14.23–18.68)	20.83 (19.89–21.78)	15.79 (13.34–16.24)
Traumatic events after the Peace Agreement***	2.52 (2.19–5.86)	2.22 (0.96–3.48)	3.18 (1.12–5.25)	4.26 (3.31–5.21)	2.80 (2.06–3.55)

*χ^2^ significant difference. *p* < 0.05.

**F(4,1164) = 50.72, *p* < 0.05.

***F(4,1164) =11.07, *p* < 0.05.

PTSD (both alone and in combination with other anxiety disorders) was by far the most common anxiety diagnosis amongst the participants. There were no gender differences in the prevalence rate of disorders, with the exception of PTSD-only; a significantly higher proportions of female participants had PTSD. There were gender differences regarding the rate of traumatic events. The mean number of traumatic events during the war was 13.14 (SD = 8.93) for women and 16.53 (SD = 9.54) for men [F (1,1168) = 36.90, p < 0.05]. The mean number of recent traumatic events was 2.08 (SD = 5.35) for women and 2.97 (SD = 6.41) for men [F (1,1165) = 6.42, p < 0.05].

The rate of some of the disorders differed significantly across rural/urban residency; the rate of GAD-only among urban residents (12.1%) was significantly higher than among rural residents (5.7%). Similarly, a significantly higher percentage of participants from urban settings (27.3%) had PTSD-only diagnoses than did rural residents (19.2%). The results of ANOVA tests showed that the mean number of traumatic events (both during the war and after the Peace Agreement) were significantly different across the different diagnostic groups.

The results of the hierarchical regression analyses are shown in Table 2. Four separate regression analyses were conducted, each with a specific anxiety disorder as the dependent variable. Table 2 also shows the results of the regression analyses for men and women. The final analysis showed that urban residency increased the likelihood of having a GAD-only diagnosis among men. Similarly, being moderately or severely socio-economically disadvantaged increased the odds ratio of having this diagnosis. For women, only being severely socio-economically disadvantaged was significantly associated with having GAD-only. Traumatic experiences were not significantly associated with having GAD-only for men or women.

**Table 2:** Results of multivariate logistic regression analysis for various anxiety disorders for men and women

**GAD-only**				
	**Male**		**Female**	
	**Model 1**	**Model 2**	**Model 1**	**Model 2**
	**Adjusted odds ratio (95% CI)**	**Adjusted odds ratio (95% CI)**	**Adjusted odds ratio (95% CI)**	**Adjusted odds ratio (95% CI)**
Age	1.01 (0.98–1.03)	1.01 (0.98–1.03)	1.02 (0.98–1.05)	1.02 (0.98–1.05)
Rural/urban Ref.: Rural	2.56 (1.32–4.97)*	2.88 (1.35–6.11)*	0.98 (0.42–2.68)	0.85 (0.34–2.16)
Socio-economic position: Mildly disadvantaged	1	1	1	1
Socio-economic position: Moderately disadvantaged	4.51 (1.35–15.04)*	4.69 (1.40–15.75)*	1.78 (0.37–8.45)	1.76 (0.37–8.45)
Socio-economic position: Severely disadvantaged	4.64 (2.08–10.37)*	4.85 (2.14–10.95)*	3.49 (1.03–11.86)*	3.53 (1.03–12.11)*
Trauma exposure during the war	–	1.17 (0.93–1.48)	–	0.75 (0.53–1.06)
Trauma exposure after the Peace Agreement	–	0.96 (0.94–1.03)	–	1.02 (0.96–1.91)
**PD-only**				
Age	0.99 (0.96–1.01)	0.98 (0.95–1.02)	0.99 (0.95–1.03)	0.99 (0.65–1.03)
Rural/urban Ref.: Rural	1.18 (0.51–2.68)	1.03 (0.40–2.65)	0.76 (0.27–2.12)	0.33 (0.89–1.22)
Socio-economic position: Mildly disadvantaged	1	1	1	1
Socio-economic position: Moderately disadvantaged	1.57 (0.42–5.81)	1.58 (0.43–5.86)	1.64 (0.31–8.62)	1.31 (0.23–7.52)
Socio-economic position: Severely disadvantaged	1.31 (0.65–2.65)	1.36 (0.67–2.78)	2.25 (0.69–8.44)	2.85 (0.80–10.01)
Trauma exposure during the war	–	1.05 (1.04–31.35)*	–	1.23 (0.93–1.33)
Trauma exposure after the Peace Agreement	–	1.02 (0.96–1.07)	–	1.11 (1.04–1.20)*
**PTSD-only**				
Age	0.98 (0.96–1.03)	0.99 (0.97–1.00)	1.02 (0.98–1.04)	1.03 (0.98–1.05)
Rural/urban Ref.: Rural	1.12 (0.67–1.87)	1.61 (0.85–2.06)	1.29 (0.73–2.28)	3.46 (1.48–8.09)*
Socio-economic position: Mildly disadvantaged	1	1	1	1
Socio-economic position: Moderately disadvantaged	2.18 (1.01–4.71)*	2.35 (1.10–5.16)*	4.52 (2.09–9.74)*	5.26 (2.32–11.92)*
Socio-economic position: Severely disadvantaged	0.82 (0.54–1.27)	0.96 (0.61–1.50)	1.40 (0.75–2.59)	1.89 (1.08–3.66)*
Trauma exposure during the war	–	1.28 (1.10–1.49)*	–	1.46 (1.21–1.76)*
Trauma exposure after the Peace Agreement	–	1.08 (1.02–1.09)*	–	1.12 (1.06–1.18)*
**Any anxiety disorder (not PTSD)**				
Age	0.99 (0.98–1.01)	0.99 (0.98–1.10)	0.99 (0.97–1.02)	0.99 (0.96–1.02)
Rural/urban Ref.: Urban	2.55 (1.61–4.04)*	2.58 (1.53–4.34)*	1.18 (0.66–2.08)	1.05 (0.55–1.98)
Socio-economic position: Mildly disadvantaged	1	1	1	1
Socio-economic position: Moderately disadvantaged	0.92 (0.38–2.19)	0.93 (0.38–2.22)	1.44 (0.55–3.72)	1.41 (0.54–3.71)
Socio-economic position: Severely disadvantaged	1.43 (0.94–2.15)	1.45 (0.96–2.20)	2.28 (1.13–4.60)*	2.30 (1.12–4.69)*
Trauma exposure during the war	–	1.08 (1.02–1.25)*	–	0.87 (0.58–1.01)
Trauma exposure after the Peace Agreement	–	1.10 (1.03–1.21)*	–	1.02 (1.01–1.07)*

*p < 0.05.

The pattern of association between trauma exposure and PD-only differed between men and women. For women, the likelihood of having a PD diagnosis increased with increasing number of recent traumatic events. For men, there was an increase in the odds ratio for PD as the number of traumatic events during the war increased.

The extent of exposure to traumatic events during the war and after the Peace Agreement was significantly associated with having PTSD-only when other variables were controlled for. For men, every additional trauma event (during the war) increased the odds of having PTSD-only by 28 percent. For women, the odds of having a PTSD-only increased by 46 percent for every additional (during the war) trauma event.

In addition, socio-economic disadvantage and urban residency increased the likelihood of having a PTSD-only diagnosis for both men and women.

When we examined the factors associated with having “any anxiety disorder” (encompassing at least one of the diagnoses of GAD, PD, OCD, agoraphobia or social phobia), exposure to trauma elevated the likelihood of having at least one anxiety disorder. For men, both older and recent trauma increased the risk of having an anxiety disorder, but for women, only the recent trauma exposure was significantly associated with having an anxiety disorder.

When considering the pattern of risk factors associated with different anxiety disorders between men and women, the impact of exposure to trauma emerged as significant for all the diagnostic groups with the exception of GAD-only.

Table 3 shows the degree of psychological distress in five different groups: GAD-only, PD-only, and PTSD-only, “any anxiety disorder”, and participants with none of the mentioned conditions (“no anxiety disorder”) for men and women. Results of one-way ANOVA showed that differences in the mean GHQ score for each of the diagnoses were statistically significant: individuals with PTSD-only were likely to have greater GHQ scores (higher degrees of psychological distress) than participants with other anxiety disorders. The GHQ scores of participants with other anxiety disorders (GAD-only, PD-only, or at least one of the diagnoses of GAD, PD, OCD, agoraphobia or social phobia were also significantly higher than those of participants with no anxiety diagnosis. Women’s scores on the GHQ-28 (mean score = 7.38, CI: 6.99–7.77) were significantly higher than men’s scores (mean score = 6.0, CI: 5.70–6.31). A Tukey post-hoc test revealed that the level of psychological distress was significantly higher for PTSD-only group than the other conditions. The differences among the levels of psychological distress in various groups were statistically significant with exception of the level of psychological distress between GAD-only and “at least one anxiety”, and between PD-only and “at least one anxiety”.

**Table 3:** Results of one-way ANOVA: differences in psychological distress (measured by mean GHQ scores) for five different diagnostic groups

	**GHQ score, Mean (95% CI)**		
	**Total**	**Men**	**Women**
GAD-only	6.21 (5.48–6.94)	6.20 (5.14–7.26)	6.35 (5.23–7.47)
PD-only	7.34 (6.34–8.35)	7.04 (5.72–8.35)	8.40 (6.50–10.31)
PTSD-only	10.09 (9.60–10.60)	9.05 (8.30–8.81)	11.10 (10.47–11.73)
“Any anxiety disorder”	6.16 (5.55–5.76)	6.15 (5.38–6.93)	6.18 (5.15–7.22)
“No anxiety disorder”	4.89 (4.26–5.15)	4.59 (4.27–4.90)	5.37 (4.90–5.83)
	F (4,1166) = 13.46, *p* < 0.05	F (4,641) = 40.60, *p* < 0.05	F (4,491) = 56.24, *p* < 0.05

## Discussion

We aimed to examine the prevalence rate and association of anxiety disorders (beyond PTSD) with trauma exposure in post-conflict South Sudan. By investigating multiple diagnoses within the same study, we were able to examine negative outcomes, other than PTSD, associated with traumatic exposure. In addition, we studied various patterns of psychological distress related to each diagnosis among men and women.

We have previously reported on the high prevalence rate of PTSD in this population [35]. In addition to a high rate of PTSD among the participants, the results of the present study showed high rates of other anxiety disorders (GAD: 15.8% and PD: 13.2%). The most salient finding of the present study is the significant association between exposure to traumatic events and anxiety disorders: exposure to traumatic events was not only associated with PTSD, but also remained as a risk factor for having PD and having “any anxiety disorder”. Trauma exposure was, however, not significantly associated with GAD in our study. Participants’ socio-economic position was also significantly associated with anxiety disorders, including PTSD. There was no significant gender difference in the prevalence rate of anxiety disorders in our study. However, gender differences were observed in the patterns of risk factors for all diagnostic groups.

The reported prevalence of DSM-IV anxiety disorders varies in the literature. The 12-month prevalence of all DSM-IV anxiety disorders is reported to range from 18.2% in the USA to 3.3% in Nigeria [49]. A recent population-based epidemiological survey in rural Kenya [50], using the Clinical Interview Schedule-Revised, reported the rates of GAD and PD to be 1.6% and 2.6%, respectively, while a South African household survey study [51], applying the World Health Organization Composite International Diagnostic Interview, showed the rates to be 2.7% and 1.2%, respectively. Our estimated rates of anxiety disorders are in accordance with other studies from conflict and post-conflict settings. For instance, de Jong et al. [6] reported prevalence rates of anxiety disorder in several post-conflict communities: 37.2% in Algeria, 40% in Cambodia, 9.6% in Ethiopia, and 13.5% in Palestine. In that study, anxiety disorder included agoraphobia and social and specific phobias, in addition to PD. A longitudinal study [52] on the consequences of the 2009 Israel–Gaza war among Israeli civilians reported the rates of GAD to range from 57.8% during the war to 21.5% and 12.6% two months and four months after the ceasefire, respectively. Data on the estimated rates of specific anxiety in low-income, post-conflict populations are very limited. Bearing in mind the variation between the study populations, and the application of a different methodology and instruments, direct comparison between the anxiety rates in our study and those of previous studies is problematic.

A higher rate of trauma experience has been identified as a risk factor for anxiety disorders in most of the limited number of studies in conflict and post-conflict settings [6,19]. A study from Nepal [53] indicated a dose–response effect of number of types of conflict events on anxiety. However, another study among the displaced population in Nepal [54] did not show any significant association between trauma exposure and anxiety symptomatology.

The patterns of association between older and recent trauma events, on the one hand, and PD-only and “any anxiety disorder” were differed across genders. Recent traumatic experiences were associated with “any anxiety disorder” among women, while both older and recent trauma remained significantly associated with having an anxiety disorder for men. Regarding PD-only, men with higher rate of trauma exposure during the war were mere likely to have PD-only. Conversely, women with higher rate of recent trauma were more likely to have a PD-only diagnosis. One explanation for this timing difference may be the difference in type of trauma events men and women are exposed to. Men and women may also experience same type of traumatic event differently: Tolin and Foa’s review [32] showed that when men and women experienced the same category of traumatic event, there was a greater frequency or severity of PTSD among women compared with men. To our knowledge, no data are available on the association between trauma exposure and the specific anxiety disorders of GAD and PD from low-income, post-conflict populations.

The association of “socio-economic disadvantage” with the anxiety disorders found in our study is consistent with findings from other studies: Socio-economic factors, such as low income and assets, unemployment, residential status, low education level, living conditions and insecurity, are known to be associated with poor general psychological health of populations affected by armed conflict [55].

Differences were observed in the rates of diagnoses across rural/urban residency. One explanation may be the fact that urban areas in South Sudan have been more affected by the recent demographic changes: the urban areas have hosted large groups of returnees since the signing of the Peace Agreement in 2005. Many are demobilised soldiers or were displaced within South Sudan and attracted to rural areas [56]. Major inequalities in health-care access and utilization are reported in Africa and low-income countries elsewhere [19]. A study from South Sudan [56] suggested that urban areas with relative availability of basic services such as healthcare, which are largely unavailable in rural areas, encourage influx from rural areas. The way these trends may influence the rate of anxiety disorders among urban population in South Sudan is however, unknown. More research is required on urban/rural health and availability, and the utilization of mental health services in the South Sudan context.

The high level of psychological distress associated with PTSD is common in the conflict-affected population [54,57-59]. The participants with PTSD in our study also showed an elevated rate of psychological distress. Another particularly important finding was the high level of psychological distress among participants with GAD-only and PD-only.

Compared with men, women had a higher rate of PTSD-only, which is in accordance with the results of other studies [32]. It is noteworthy that the influence of gender was not a statistically significant in the prevalence of GAD and PD. In most studies of low-income, conflict-affected populations, women have been found to have poorer general psychological health [55] and reported higher rates of anxiety [17,53]. Other studies [50,60] have failed to report gender differences in the prevalence of common mental disorders. Similarly, in a study of a natural disaster-affected population in Vietnam [10], gender was not a risk factor for GAD or PD. Gender differences were, however, observable in the degree of psychological distress associated with anxiety disorders. Compared with men, women reported a higher degree of psychological distress, which is consistent with the findings of other studies [32].

Despite the challenges of carrying out research in post-conflict settings [61], this study illustrates the feasibility of conducting a community survey in the post-conflict setting of South Sudan. There were several challenges associated this study, such as a lack of proper infrastructure, which made it difficult to reach some of the sampling areas. Another challenge was ensuring that the survey was conducted in a safe environment, which required careful and continuous monitoring of the situation. The survey had a high response rate, which was partly due to the community leaders’ approval of the study. The socio-demographic characteristics of our sample were comparable to those of the general population and similar to socio-demographic characteristics found in the study by Roberts et al. in Juba [34].

This study has several potential limitations. The 2008 Sudan census, which was used as the source of population data and in the sampling process, has inaccuracies, particularly because of the large-scale migration process and the influx of returnees. In addition, the a priori exclusion of the insecure areas creates a bias that is difficult to estimate. As a cross-sectional study, it cannot identify cause-and-effect relationships between traumatic exposure and anxiety diagnoses.

A further limitation is that self-reported measures were used to assess exposure to traumatic events; inconsistencies in the recall of events may introduce a bias [62]. Self-reported measures rely on the participant’s memory and are prone to the influence of dominating attitudes towards the themes of the study. The use of an additive scale of traumatic events is a simple way of including an indicator of exposure. However, this would not differentiate between the types and the severity of the events.

While the instruments used in this study have been widely and internationally used in various cultural settings, and the interviewers were familiar with the socio-cultural setting, no formal socio-cultural validation was conducted. The interviewers translated some of the words in the questionnaire into the indigenous languages, and used them in about 20% of the interviews. The use of the indigenous languages was, however, not systematically measured and hence represents a source of bias. We were not able to formally assess interrater reliability. However, an attempt was made, through repeated and supervised interview practice, to ensure a satisfactory level of rating agreement among the interviewers.

These limitations may influence the generalizability of the study results. We believe, however, that the results are relevant for Greater Bahr el Ghazal states as well as for other post-conflict settings. The findings cast light on various aspects of the war-affected society of South Sudan, indicating the similarities and peculiarities of this setting in comparison with other post-conflict societies in the impact of war on the health of the population.

## Conclusions

This study showed distress related to exposure to traumatic events in the post-conflict population extended beyond PTSD. High rates of anxiety disorders other than PTSD, and the psychological distress associated with these, have possible implications for the construction and provision of health services to individuals exposed to traumatic events. The risk factors for anxiety disorders, particularly the gender differences in the risk factor patterns, may help guide future health planning in South Sudan, and should be considered in other low-income countries. A clinical implication of this finding is that persons exposed to traumatic events should be screened not only for PTSD but also for other anxiety symptoms. The significant association of socio-demographic factors suggests mental health studies performed in post-conflict settings should be broadened to include risk factors beyond exposure to traumatic events.

## Competing interests

The authors declare that they have no competing interests.

## Authors’ contributions

TA: executed the statistical analysis and drafted the manuscript; participated in the design of study. LL: participated in the design of study and drafting of the manuscript. AHE: participated in the design of study and drafting of the manuscript. LS: participated in the drafting of the manuscript. EH: supervised, participated in the design of study and drafting of the manuscript. All authors read and approved the final manuscript.

## Pre-publication history

The pre-publication history for this paper can be accessed here:

http://www.biomedcentral.com/1471-244X/14/6/prepub
